# Refugee Status Determination Procedure and Mental Health of the Applicant: Dynamics and Reciprocal Effects

**DOI:** 10.3389/fpsyt.2020.587331

**Published:** 2021-01-20

**Authors:** Maša Vukčević Marković, Nikola Kovačević, Jovana Bjekić

**Affiliations:** ^1^Department of Psychology, Faculty of Philosophy, University of Belgrade, Belgrade, Serbia; ^2^PIN–Psychosocial Innovation Network, Belgrade, Serbia; ^3^Faculty of Law, University of Union, Belgrade, Serbia; ^4^Institute for Medical Research, University of Belgrade, Belgrade, Serbia

**Keywords:** psychological status, post-traumatic stress disorder, asylum procedure, refugees, international protection, psychological and psychiatric assessment

## Introduction

After being forced to flee their home countries, which is often preceded by traumatic experiences, refugees and asylum seekers are faced with multiple new transit risks while searching for safety (1–12). If they manage to make it through this journey, after arriving to destination countries, they must begin the long and often exhausting process of rebuilding their lives. This phase includes addressing existential concerns and reestablishing control over one's life, as well as psychological stabilization and going through the different phases of recovery from trauma. However, there is a growing evidence on the numerous challenges and risks for mental health stability and general well-being this phase can bring for a person in need of international protection.

There are numerous post-migration factors related to refugees' and asylum seekers' mental health and well-being (1, 2, 4, 6, 8, 13–20), including communication difficulties, difficulty in finding work and poor job conditions (2, 6, 14, 15, 21), low quality housing (22–25), difficulties in accessing health care and social services (14), loss of culture, limited access to traditional foods (1, 2, 14, 18), and reduction of social support networks which lead to experiences of isolation and loneliness (15, 26–29).

The refugee status determination procedure is a crucial step, and pre-condition for initiating the phase of rebuilding one's life. This procedure has been shown to have not only practical outcomes in terms of legal status determination and the rights it guarantees, but also a complex, dynamic, and reciprocal relationship with the mental health of the applicant, which carries additional protection and health risks. We see that this relationship is reflected through (1) the impact that different stages of the asylum procedure can have on mental health difficulties and well-being and (2) the impact different mental health difficulties can have on the refugee status determination procedure. Here we provide an evidence-based perspective on the reciprocal effects of the refugee status determination procedure and applicants' mental health status, with primary focus on trauma-related difficulties including post-traumatic stress disorder (PTSD); and provide arguments for increased sensitivity to mental health difficulties in refugee status determination procedure in order to minimize negative impact that the procedure may cause to the applicants' mental health as well as to reduce bias that can stem from PTSD symptomatology when making judgments the credibility of applicant testimonials.

## Refugee Status Determination Procedure: Challenges for Mental Health of the Applicant

The applicant must comply with several requirements in order to obtain international protection. One of them is a detailed report on the reasons for leaving their home country and previous experiences, which often includes reporting on traumatic experiences and painful human suffering that the person was exposed to. This process has been shown to increase the risk of both jeopardizing psychological stability and well-being and of retraumatization. These risks are even higher if the person in question is suffering from PTSD and/or is currently undergoing the phases of recovery from trauma in which ensuring a non-stressful and protective environment is of crucial importance.

Previous studies have shown that asylum interviews can have a stressful impact on traumatized refugees, indicating that asylum interviews can increase symptoms of intrusions (30). It has also been demonstrated that a longer asylum procedure and delays and uncertainties during the legal status determination process may have negative effects on refugees' psychological state and well-being (4, 6, 8, 13, 14, 20). In addition to this, the period during which they are expecting an asylum decision represents the phase in the asylum process that can trigger deterioration of psychological stability and impose additional risks for a person. We have witnessed, that in the case of a negative decision, there is an increased risk for a person to develop PTSD, depression and anxiety related difficulties, as well as suicidal ideation and intentions (31, 32). On the other hand, it has been demonstrated that obtaining international protection improves not only the overall well-being of a person, but also increases effects of trauma-focused therapy for PTSD (33).

It should also be noted that plethora of factors can mediate the effects of the status determination procedure on mental health. These factors include, but are not limited to gender, age, education, economic resources, country of origin, cultural, or religious background as well as previous traumatic experiences of war, torture, and family separation (18). All these can serve as both additional risk- and protective-factors depending on the individual circumstances (e.g., some age groups may be more vulnerable, but in response to that may have access to more focused and specialized services).

## Psychological State of an Applicant: Challenges for Refugee Status Determination Procedure

Trauma-related psychological difficulties a person in need for international protection can experience represents an additional challenge in this process which can affect different stages of asylum procedure. Namely, the decision to apply for the asylum, the preparation for the asylum interview, the hearings i.e., asylum interviews, determination process, and finally the asylum decision can all be affected by applicants' psychological state. Here we focus predominantly on the asylum interviews as the stage where bias due to mental health is the most likely to happen, and with the most serious ramifications. Due to limited scope of this paper, we showcase how mental health may affect status determination procedure using the example of PTSD symptomatology.

People suffering from PTSD will experience at least some symptoms from a cluster of *Persistent avoidance of stimuli associated with the traumatic event(s)*, indicating that a person will tend to *avoid distressing memories, thoughts, or feelings about or closely associated with the traumatic event(s)* and/or *external reminders (people, places, conversations, activities, objects, situations) that arouse distressing memories, thoughts, or feelings about or closely associated with the traumatic event(s)* (34). This can affect both readiness to apply for international protection, since applying for asylum would require a discussion of the trauma details (35), as well as difficulties in proceeding through the long and exhausting asylum procedure consisting of several stages which require a person to speak about traumatic experiences not only in detail but also repeatedly, e.g., on several occasions in asylum hearings.

Understanding the *negative alterations in cognitions and mood associated with the traumatic event(s)*, cluster of PTSD symptoms (34) is of particular importance for mitigating its potential effects on the refugee status determination procedure. Namely, this process is based on appropriate evidence, but also to a large extent, determined by the capacities of the person and available legal aid which can provide support through the process of attaining credibility and making one's testimony believable Two aspects of this process are of particular importance due to their links with the psychological state of the person and the way in which trauma can affect one's capacities for providing believable/credible testimony. Specifically, people suffering from PTSD may experience difficulties related to an *inability to remember an important aspect of the traumatic event(s)*, which is exactly what is requested of an applicant during the asylum procedure. This could result in discrepancies in statements or an inability to recall some details of the traumatic experience which could be of crucial importance for the asylum claims. Previous studies indicated that these discrepancies can to occur in repeated asylum interviews and that for asylum seekers with severe post-traumatic stress, the number of discrepancies increased with the length of time between interviews (36). Results of this study strongly suggest that the assumption that discrepancies in statements or the inability to recall details of traumatic events reflect poor credibility should be put in question.

In addition to implications related to the cognitive aspect and verbal statements, the same cluster of symptoms is also related to one's emotional reactions, and can be experienced as *persistent negative emotional state, feelings of detachment or estrangement from others or persistent inability to experience positive emotions* (34), which can result in the absence of emotional reactions that are expected to follow different verbal statements and, therefore, the potential applicant may not display what would be considered normal emotional responses while recalling a traumatic event (35). Thus, the expectation that a person speaking about terrifying suffering must, at least to a moderate extent, demonstrate visible distress can be misleading and result in an incorrect conclusion that the absence of such reactions indicates the questionable credibility of asylum claims. It is, therefore, important to bear in mind that if a person is feeling emotionally numb, or if they experience a general lack of emotional reactions, this could be a consequence of trauma-related psychological difficulties and should be carefully considered during the asylum procedure.

Finally, people experiencing PTSD, or other trauma-related difficulties, will experience at least some of the difficulties related to *marked alterations in arousal and reactivity associated with the traumatic event(s)*, including *hypervigilance* and *problems with concentration and sleep*, which could impact the asylum procedure and make it even more challenging for both the applicant and representatives of the decision-making authority.

## Implications and Action Points Needed

Bearing in mind the aforementioned challenges and links between the refugee status determination procedure and the psychological state of the applicant, action points and recommendations that lead to the prevention of both deterioration of applicant's mental health and unbiased, trauma-informed asylum decisions we believe need to be carefully considered. The issues of mental health assessment, as well as methodological and ethical considerations in designing refugee studies have been discussed in detail elsewhere (37–43), therefore here we focus on practical implications for policy makers and practitioners to build upon this evidence and establish data-driven approach to mental health protection during different stages of refugee status determination procedure.

It is important to outline that asylum procedures across the Europe are regulated in a different manner, and in terms of its stages, authority competent to decide on asylum claims, availability of legal, or psychosocial support at different stages, length of procedure, differential treatment of certain nationalities, etc. (44). However, the legal solutions do not always reflect the state of affairs in practice, which might impact asylum applicants and their expectations that are based on their knowledge on the existing legal system. Thus, legal aid, but also psychological support can be extremely significant for an individual who might be informed on the law and the steps in the procedure, but unaware of the practice. For instance, different European states have different time limits for the first instance procedure. It can last from 8 working days (45), to up to 21 months (46). Also, due to a high number of applicants, legal deadlines are often breached (47), sometimes even significantly, or the length can depend on the nationality of the applicant (48).

Therefore, it is of crucial importance, especially in countries that are developing and adjusting their asylum procedures, to establish multidisciplinary teams which will enable sensitive preparation for the asylum procedure by providing relevant information, continuous support throughout its different stages and, if needed conducting interventions by mental health experts after the interviews or hearings in order to prevent the deterioration of applicant's mental health and well-being. These teams should, by using different perspectives and expertise, be able to identify a wide scope of potential risks and intervene in a timely manner in order to provide proper protection and support.

Moreover, training programs aiming to educate and sensitize both legal representatives and decision-makers should be introduced and continuously implemented. These programs should help practitioners and decision-makers to recognize signs of psychological vulnerability and understand the effects PTSD, and other psychological difficulties could have on the asylum procedure. Finally, training programs should lead to a better understanding of the needs of traumatized refugees during asylum interviews and hearings which could lead to the asylum determination process becoming more mental health sensitive, resulting in readiness of relevant practitioners, and decision-makers to carefully consider total length of the asylum process, duration of asylum interviews, and hearings and the risks for retraumatization or jeopardizing psychological stability of an applicant. These measures can not only protect the mental health and well-being of a person in need of international protection, but also improve the quality of the decision-making process in the refugee status determination procedure.

## Author Contributions

MV drafted the article. NK and JB critically reviewed and revised the article. All authors contributed equally to the conception, design of work, read and approved the final version submitted for publishing.

## Conflict of Interest

The authors declare that the research was conducted in the absence of any commercial or financial relationships that could be construed as a potential conflict of interest.
